# MVI-Mind: A Novel Deep-Learning Strategy Using Computed Tomography (CT)-Based Radiomics for End-to-End High Efficiency Prediction of Microvascular Invasion in Hepatocellular Carcinoma

**DOI:** 10.3390/cancers14122956

**Published:** 2022-06-15

**Authors:** Liyang Wang, Meilong Wu, Rui Li, Xiaolei Xu, Chengzhan Zhu, Xiaobin Feng

**Affiliations:** 1 School of Clinical Medicine, Tsinghua University, No. 1 Tsinghua Yuan, Haidian District, Beijing 100084, China; wly21@mails.tsinghua.edu.cn (L.W.); wmliliver@163.com (M.W.); xuxl21@mails.tsinghua.edu.cn (X.X.); 2 Department of Hepatobiliary and Pancreatic Surgery, The Affiliated Hospital of Qingdao University, No. 16 Jiangsu Road, Qingdao 266003, China; lr022319@163.com; 3 Hepato-Pancreato-Biliary Center, Beijing Tsinghua Changgung Hospital, School of Clinical Medicine, Tsinghua University, No. 168 Litang Road, Changping District, Beijing 102218, China

**Keywords:** microvascular invasion, radiomics, end-to-end, deep learning, clinical decision

## Abstract

**Simple Summary:**

Microvascular invasion is an important indicator for reflecting the prognosis of hepatocellular carcinoma, but the traditional diagnosis requires a postoperative pathological examination. This study is the first to propose an end-to-end deep learning architecture for predicting microvascular invasion in hepatocellular carcinoma by collecting retrospective data. This method can achieve noninvasive, accurate and efficient preoperative prediction only through the patient’s radiomic data, which is very beneficial to doctors for clinical decision making in HCC patients.

**Abstract:**

Microvascular invasion (MVI) in hepatocellular carcinoma (HCC) directly affects a patient’s prognosis. The development of preoperative noninvasive diagnostic methods is significant for guiding optimal treatment plans. In this study, we investigated 138 patients with HCC and presented a novel end-to-end deep learning strategy based on computed tomography (CT) radiomics (MVI-Mind), which integrates data preprocessing, automatic segmentation of lesions and other regions, automatic feature extraction, and MVI prediction. A lightweight transformer and a convolutional neural network (CNN) were proposed for the segmentation and prediction modules, respectively. To demonstrate the superiority of MVI-Mind, we compared the framework’s performance with that of current, mainstream segmentation, and classification models. The test results showed that MVI-Mind returned the best performance in both segmentation and prediction. The mean intersection over union (mIoU) of the segmentation module was 0.9006, and the area under the receiver operating characteristic curve (AUC) of the prediction module reached 0.9223. Additionally, it only took approximately 1 min to output a prediction for each patient, end-to-end using our computing device, which indicated that MVI-Mind could noninvasively, efficiently, and accurately predict the presence of MVI in HCC patients before surgery. This result will be helpful for doctors to make rational clinical decisions.

## 1. Introduction

Hepatocellular carcinoma (HCC) is a major histological subtype of liver cancer, accounting for 90% of primary liver cancers, and the third most common cause of cancer-related mortality worldwide [1,2,3]. It is one of the most common malignant tumors worldwide, especially in Asia, Africa, and southern Europe. Genetics; epigenetic changes; chronic hepatitis B and hepatitis C virus infections; and unhealthy lifestyle habits, such as smoking, irregular diet, and sleep deprivation are the main risk factors for liver cancer [4,5,6]. The early clinical symptoms of HCC are not obvious and mainly include liver pain, fatigue, weight loss, low-grade fever, and loss of appetite. However, ascites, jaundice, anemia, weight loss, and cachexia occur in the later stages. Additionally, complications such as hepatic encephalopathy and tumor rupture can be induced [7]. The clinical decisions made for individual patients with HCC greatly affect their prognosis. At present, the most recognized treatment options are surgical resection [8] and liver transplantation [9], but serious challenges remain in achieving precise planning for treatments. Therefore, it is of great value for the treatment of HCC to make full use of clinical information, such as radiological data before surgery.

At present, various clinical studies on HCC have been conducted, which are helpful for the diagnosis and treatment of HCC. For example, studies have shown that gadolinium magnetic resonance can enhance the sensitivity of noninvasive diagnoses for hepatocellular carcinoma nodules in patients with liver cirrhosis, which is beneficial to the early diagnosis of HCC [10]. Moreover, the latest Liver Imaging Reporting and Data System (LI-RADS) classification was validated as a potential and readily applicable predictor of HCC pathological features and tumor histology, and patient clinical characteristics had a significant impact on postoperative recurrence outcomes [11]. The drug treatment of HCC has also progressed, such as the use of regorafenib to obtain significant efficacy [12].

Microvascular invasion (MVI) in HCC refers to the nests of cancer cells (more than 50 cancer cells) observed under a microscope in a vascular lumen lined by endothelial cells, and it is an important reason for the poor long-term survival rate after HCC surgery [13]. MVI is most common in the small branches of the portal vein in adjacent tissue, followed by the branches of the hepatic vein, hepatic artery, bile duct, and lymphatic vessels. Several studies have shown that MVI is an independent factor for postoperative tumor-free survival, and it can be used to effectively predict intrahepatic metastasis, multi-nodular recurrence, and can significantly reduce survival [14,15,16]. Adequate surgical margins such as anatomical resection are an important methods for reducing postoperative recurrence rates, which allow complete resection of the tumor-bearing portal vein branches, resulting in more efficient eradication of intrahepatic MVI [17,18]. However, a large amount of liver tissue needs to be removed during the operation, which leads to a high possibility of postoperative liver insufficiency. Therefore, large-scale liver tissue removal is costly.

It is worth noting that the clinical method that is generally used for diagnosing MVI is postoperative pathological detection, wherein the tissue specimens taken during the operation are observed under a microscope; this is accurate but not helpful for preoperative clinical decision making [19]. Preoperative prediction of MVI can help to guide surgical strategies for liver transplantation and hepatectomy. For example, doctors would know in advance whether a patient has MVI; this would help them to formulate precise surgical strategies. Early prediction of MVI is also beneficial for doctors to take measures to prevent recurrence and metastasis before surgery, including systemic therapy or immunotherapy [20]. Therefore, the use of cutting-edge technologies to develop preoperative noninvasive MVI prediction tools can better guide clinical decision making in HCC patients.

With the development of artificial intelligence (AI) technology, radiomics and machine learning methods have gradually been applied to the preoperative prediction of MVI in liver cancer, and excellent performance has been achieved [21,22]. For example, Jiang et al. [23] included a study of 405 HCC patients and extracted 7302 radiomic features for predicting MVI from their radiomics data. The area under the receiver operating characteristic curve (AUC) of the extreme gradient boosting (XGBoost) algorithm reached 0.887. Nebbia et al. [24] retrospectively collected preoperative multiparametric liver magnetic resonance imaging (MRI) scans from 99 HCC patients and regions of interest (ROI) were manually segmented by radiologists. First, the researchers extracted radiomic features in the region, which were then fed into a machine learning model for predicting MVI. The best performance was obtained when a combination of multiple MRI sequences was used, with an AUC of 0.8669. In recent years, deep learning technology has gradually emerged and has been applied to various medical tasks. In related studies on the preoperative prediction of MVI, this technique has also been shown to have a stronger generalization ability. Some researchers constructed a 3D convolutional neural network (-CNN), which was able to perform preoperative diagnosis of MVI in HCC patients by inputting MRI sequences, with the highest AUC of 0.81 and a sensitivity of 0.69 [25]. In addition, a 2D-CNN model was applied to this task [26] and showed excellent performance. However, it should be emphasized that most published studies have required the help of experienced radiologists to manually segment the ROI or volume of interest (VOI), which is inefficient and cannot be automated and batched. Developing an end-to-end deep learning method that integrates raw data preprocessing, automatic ROI segmentation, and MVI prediction is conducive to promotion.

In this study, we propose an end-to-end deep learning strategy for preoperative MVI prediction, named MVI-Mind. It can accurately predict the presence of MVI with only the input of raw computed tomography (CT) images of patients with HCC. The transformer architecture in the field of natural language processing (NLP) was introduced into the segmentation of liver tumors and surrounding tissues, and the effect was better than that of other supervised learning segmentation algorithms. An efficient convolutional neural network (CNN) model was designed to achieve automatic feature extraction and prediction. To the best of our knowledge, this is the first report of an end-to-end deep learning method that integrates raw data preprocessing, automatic ROI segmentation, and MVI prediction.

## 2. Materials and Methods

**Figure 1:**
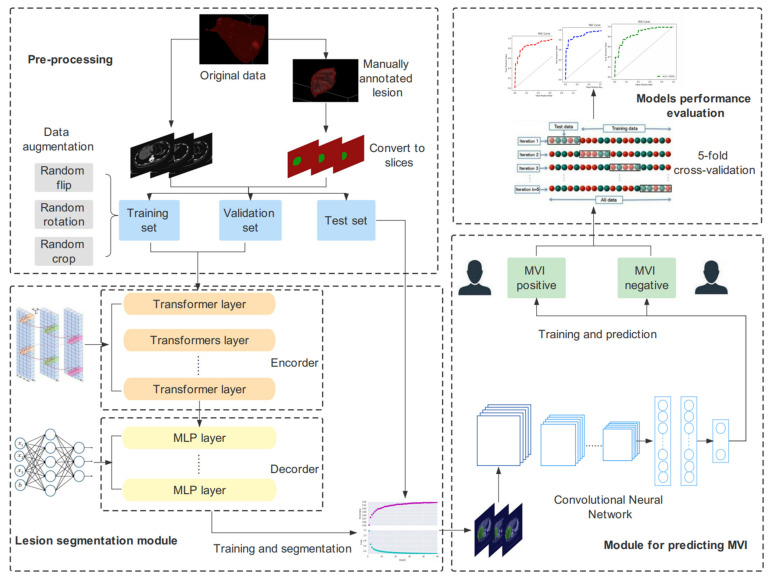
The workflow of this study.

The deep learning strategy proposed in this study included four modules: data preprocessing, ROI segmentation module, MVI prediction module, and method evaluation and comparison. The workflow is illustrated in Figure 1. Preprocessing included manual annotation, data cropping, image dimension transformation, dataset partitioning, and data augmentation. The segmentation module adopted a lightweight transformer supervised learning algorithm, which made it more suitable for the segmentation of liver tumors and surrounding tissues. An efficient CNN was designed in the prediction module to extract the features of the segmented images and to perform accurate classification and was also compared with other deep learning models to evaluate the superiority of the proposed method.

### 2.1. Patients

In this study, we followed the principles of the Declaration of Helsinki and the study was approved by the hospital ethics committee (ethics number 20001-01). All patients provided informed consent before surgery. The project was registered in the National Hepatobiliary Standard Database of China (registration number CDR/20221019).

**Figure 2:**
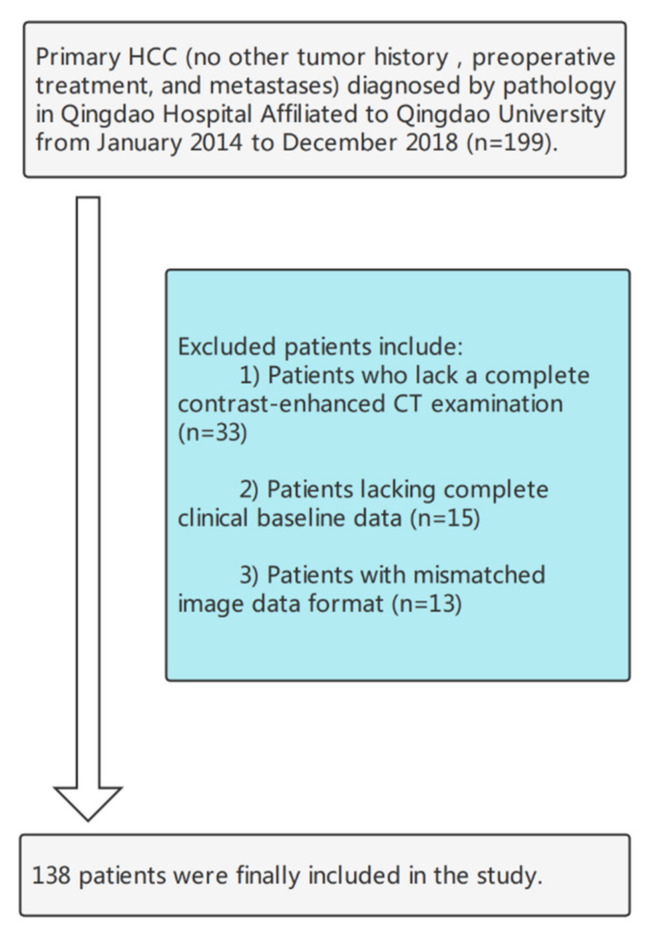
The flowchart of the patient selection process.

Data of patients with HCC who underwent liver surgery at Qingdao University between January 2014 and December 2018 were retrospectively collected. Tumor specimens from each patient underwent postoperative pathological examination for MVI. The inclusion criteria were as follows: (1) HCC was diagnosed based on pathology; (2) partial hepatectomy was the first treatment, and (3) contrast-enhanced CT examination was performed within 1 month before surgery, and all periods were complete. Patients were excluded from the study based on the following criteria: (1) chemotherapy, interventional therapy, targeted therapy, and other treatments before partial hepatectomy; (2) history of other tumors; (3) incomplete imaging and clinical medical records; (4) lesion had metastasized. Ultimately, 138 patients were selected for the study and their radiological and clinical data were obtained. The patient selection process is shown in Figure 2.

### 2.2. Imaging Acquisition and Preprocessing

The scanning equipment used in this experiment was a German CT (SOMATOM Definition Flash, Siemens) and an American Discovery CT (GE Healthcare). The scan was performed as a three-level contrast-enhanced scan of the upper abdomen, ranging from the top of the liver to the lower edges of the two kidneys. Scanning parameters included a voltage of 120 kV, current of 200–350 mA, scan layer thickness of 5 mm, layer spacing of 5 mm, and matrix size of 512 × 512. Furthermore, iohexol and 350 mg/m1 of iodine were injected via a peripheral vein at a flow rate of 3.0 mL/s and a dose of 1.5 mL/kg using a pressure syringe. The arterial phase (AP), portal venous phase (PVP), and equilibrium phase (EP) delays were 30 s, 60 s, and 120 s, respectively. AP, PVP, and delay period (DP) images were obtained.

Typically, CT scans store raw voxel intensities in Hounsfield units (HU). In this study, the CT scans were normalized with thresholds of −1000 and 400 (normalization). The original data contained many slices without an ROI, which increased the amount of unnecessary computation. The slices were searched from the beginning to the end of the ROI based on manually annotated data (mentioned below), and the rest were cropped. The input channel of the deep learning framework designed in this paper was 2-dimensional; therefore, the data were converted to the corresponding format. Additionally, the dataset was divided before the automatic segmentation and MVI prediction tasks, in which the training set performed data augmentation operations to balance the data categories, including horizontal flipping, random rotation, and random blurring [27].

### 2.3. Manual ROI Annotation

MVI-Mind employed supervised learning to train the segmentation model, which means that CT images must be manually labeled with ROI first. In this study, all CT data from 71 patients were manually labeled by two radiologists, each with more than 10 years of experience, considering the liver tumor and parts of the surrounding tissue (extending 1 cm from the tumor boundary). One physician independently used the 3D Slicer (Boston, MA, USA) software to delineate the ROI of each slice and finally formed a VOI. Another physician reviewed the marked results and accepted them if there were no disputes. It is worth noting that, in this study, we did not manually delineate the data of all patients, which reduced the huge workload and highlighted the superiority of deep learning segmentation, i.e., the automatic segmentation of all data with a limited number of labels.

### 2.4. The Construction of the Segmentation Models

#### 2.4.1. Transformer-Based Lightweight Design

Transformer-based neural networks have been used in the NLP field since 2018 and have achieved remarkable results as compared with recurrent neural networks (RNNs) [28]. This architecture proposes a way to process sequential data in parallel, and therefore, it is much faster than previous architectures, and it is also excellent at handling long-term dependencies. A self-attention mechanism was used to capture contextual information better. Transformers have been applied in the field of computer vision since 2021. Surprisingly, it surpassed CNN in tasks such as image classification, semantic segmentation, and object detection, becoming the most promising neural network [29,30,31].

However, traditional transformers have a large number of parameters and are difficult to train, which puts high demand on computers. Based on this, for MVI-Mind, referring to [32], we proposed a lightweight transformer architecture for automatic segmentation of the liver ROI, which reduced the training difficulty, and also had better performance. To the best of our knowledge, this is the first study to adopt this architecture for segmentation of HCC lesions.

**Figure 3:**
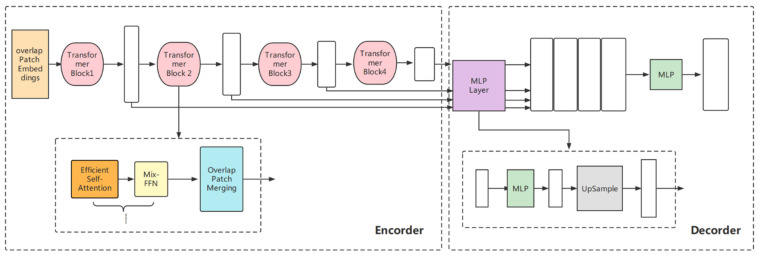
Proposed lightweight transformer architecture.

Figure 3 is a schematic diagram of the architecture of the model, including a novel hierarchical transformer encoder, which outputs multiscale features and does not require positional embedding. This avoids the difference in resolution during testing and training and results in performance degradation. The decoder adopts a lightweight multilayer perceptron (MLP) decoder to aggregate information from different layers, thereby combining the local and global attention. Specifically, the encoder removes the traditional positional embedding and replaces it with a mix-feed-forward network (Mix-FFN), which introduces a 3 × 3 depthwise convolution in the feed-forward network to transfer positional information. The decoder only introduces several MLP layers and does not perform complex operations, which significantly reduces the number of parameters and computations. Moreover, for the patch embedding of the network, the patch with the overlap operation is designed such that the non-independent design can ensure local continuity. For traditional self-attention, we also refer to [32] to employ efficient self-attention, which mainly increases the hyperparameter sr_ratio based on the original to control the size of the parameter matrix.

In this study, the transformer architecture only allows the input of two-dimensional images; therefore, we batched the CT data in the form of slices (image size was converted to 512 × 512). To further improve the generalization ability of the model, we used mix_vision_transformer_b5 as the pretraining model [32] for transfer learning, in which the backbone selected MixVisionTransformer_B5, and the embedding_dim was set to 768.

#### 2.4.2. Model Comparison—Swin Transformer

To reflect the superiority of the lightweight transformers adopted by MVI-Mind in the liver ROI segmentation task, the Swin Transformer model was selected for comparison. Swin transformers, proposed in 2021 [33], have achieved a mean intersection over union (mIoU) of 53.5 on the semantic segmentation dataset ADE20K, which once led all deep learning models.

Swin transformers adopt a common architecture based on moving panes and hierarchical representations. Moving windows limit self-attention to a certain range, which greatly reduces the amount of computation and enables interactions between nonlocal windows. Specifically, the model builds a hierarchical feature map of an image on the basis of linear computational complexity, that is, a hierarchical feature representation is constructed by merging neighborhoods layer-by-layer through patches. Such an architecture enables the model to achieve dense prediction tasks, similar to architectures such as U-Net.

It was applied to the segmentation of areas, such as liver lesions, with slices of CT data as input, and the image size was set to 512 × 512 pixels in this work. The training method also adopted transfer learning and selected the pretraining model, swin_transformer_base_patch4_window7_224_imagenet_1k. Meanwhile, we employed SwinTransformer_base_patch4_window7_224 as the backbone.

#### 2.4.3. Baseline Models

The DeepLab v3+ model used atrous convolution operations and performs well in semantic segmentation, object detection, and other fields [34]. This study selected this as the baseline model. Additionally, U-Net has been adopted as a classic model for medical image segmentation [35]. Slices of size 512 × 512 were input into the baseline models and ResNet-101 was selected as the backbone of DeepLab V3+.

### 2.5. The Construction of the MVI Prediction Models

#### 2.5.1. The Proposed CNN Model

**Figure 4:**
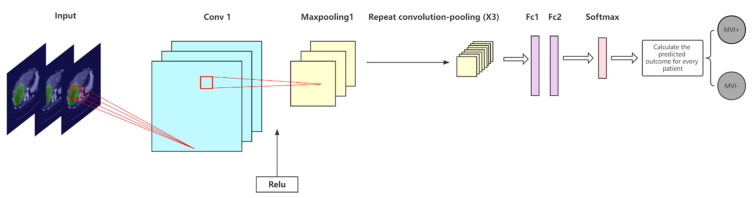
Convolutional neural network (CNN) designed to extract region-of-interest (ROI) features and make predictions in this study.

We designed a CNN architecture for the segmented ROI to efficiently and accurately predict whether a patient has MVI; its network diagram is shown in Figure 4. Four convolutional layers and four max pooling layers (the convolutional layer and pooling layer are alternately arranged), two fully connected layers, and a softmax layer are included in the model. The ReLU activation function was selected, the convolution kernel size was set to 3 × 3, the padding was 2, and the stride was 2. To prevent overfitting, a dropout technique was employed before the fully connected layers. The architecture of the CNN model proposed in this paper is relatively simple and has no redundant layers; therefore, the training difficulty is low and it can efficiently complete the MVI prediction task.

Because the segmentation module took a 2-dimensional image input, the input channel of this prediction model was also designed to be 2-dimensional, and the input image size was 512 × 512. It is worth noting that although slices of CT data were used for training, the evaluation and prediction of the model were performed on patients because the results were clinically meaningful. Because liver tumors are three-dimensional and MVI positivity may not be captured in every slice, it may not be the case that all slices are predicted to be the same. The experiments aggregated predictions across all slices for each patient and followed the clinical decision-making workflow guidelines adopted by radiologists. The guidelines state that a patient is considered to have MVI if one of the slices is positive for MVI. A patient was considered to be free of MVI only when all the slices were negative. Whether a patient is positive or not depends on the slice with the highest predicted probability. Based on this, we calculated the probability of MVI positivity for each patient. The rationality and scientificity of this calculation method are confirmed in [26].

#### 2.5.2. Comparison with Other CNN Models

Studies have been conducted using other CNN architectures to predict MVI [26,36]. Two classic models, ResNet-34 and Inception V3, were compared. With the advantage of residual learning, ResNet has performed well in many medical image-recognition tasks [37,38]. In this work, a 34-layer ResNet was built. The inception architecture was proposed by Google and has performed well in several data-mining competitions [39]. One of the improved versions, Inception V3, was adopted. The main idea of this model is to employ dense components to approximate an optimal local sparse solution. We also calculated MVI predictions for each patient using the clinical decision guidelines described above.

### 2.6. Model Evaluation Indicators

#### 2.6.1. Segmentation Models

To evaluate the segmentation module, we selected mIoU, accuracy (Acc), Kappa coefficient, and Dice similarity coefficient, and the corresponding calculation methods are shown in Equations (1)–(4). Among them, the mIoU is often adopted as a standard measure of semantic segmentation, and the other indicators can also reflect the performance of the segmentation model.

(1) $$\mathrm{mIoU}=\frac{1}{k+1}\overset{k}{\underset{i=0}{\sum }}\frac{TP}{FN+FP+TP}$$

(2) $$\mathrm{Acc}=\frac{TN+TP}{TN+TP+FN+FP}$$

(3) $$\mathrm{Kappa}=\frac{P_{0}-P_{e}}{1-P_{e}}$$

(4) $$\mathrm{Dice}=\frac{2TP}{FP+2TP+FN}$$

*TN*, *TP*, *FN*, and *FP* represent the true negative, true positive, false negative, and false positive numbers, respectively; *P*_0_ is the overall classification accuracy; and *P_e_* is the ratio of the sum of the total number of samples multiplied by the predicted number to the square of the total number.

#### 2.6.2. MVI Prediction Models

Acc, recall rate (Rec), precision (Prec), and F1 score (the corresponding calculation methods are given in Equations (2) and (5)–(7)) were selected as the evaluation indicators of the prediction models. The MVI prediction in this study was a binary classification task and the classification threshold was set to 0.5. To assess the robustness of the models, the mean and 95% confidence interval (CI) of the statistical results were calculated. Moreover, the receiver operating characteristic (ROC) curve was obtained by plotting the true positive rate (TPR) and false positive rate (FPR) under different threshold settings, which could objectively reflect the generalization ability of the model, as well as the AUC in the evaluation criteria:

(5) $$\mathrm{Rec}=\frac{TP}{TP+FN}$$

(6) $$\mathrm{Prec}=\frac{TP}{TP+FP}$$

(7) $$F1=2\cdot \frac{Prec\cdot Rec}{Prec+Rec}$$

*TN*, *TP*, *FN*, and *FP* represent true negative, true positive, false negative, and false positive numbers, respectively.

## 3. Results

### 3.1. Experimental Setup

Before performing the segmentation task in this study, the datasets of the three periods were randomly divided into training, validation, and test sets at a ratio of 8:1:1. At the end of the training, the test set was employed to evaluate the model performance. When performing the MVI prediction task, considering the small number of patient samples, the 5-fold cross-validation method was selected in this study, that is, the dataset was randomly divided into five equal parts each time, four of the parts were used for training and the remaining part was used for testing, the process was repeated five times, and finally, the mean value and the corresponding 95% CI of five results were counted. This process was performed separately for the CTs at different time periods. It must be emphasized that because the slices of the same patient may have a high degree of similarity, if all slices are divided as a whole, data leakage would occur, resulting in increased artificial precision. Therefore, we divided the patients. All the experiments were completed in the Windows 10 operating system, and the relevant computing equipment was configured with a CPU AMD Ryzen 7 5800H with 16 GB memory and two GPUs, NVIDIA^®^ GeForce RTX 3070 and NVIDIA^®^ Tesla V100 (32GB memory); both were supported by the compute unified device architecture (CUDA) GPU acceleration. All work was implemented using Python 3.8, and the PaddlePaddle deep-learning framework.

### 3.2. Statistics of Clinical Characteristics

Patient information and clinical indicators were grouped (MVI+ and MVI−) for statistical analysis, and the results are shown in Table 1. Patient information included sex, age, and other clinical test indicators, including tumor size, tumor markers, and liver function indicators. Most patients were men, with an average age of 56 years. Notably, the maximum tumor diameter in MVI-positive patients was significantly larger than that in MVI-negative patients (*p* = 0.0321). Additionally, the proportion of alpha-fetoprotein (AFP) positivity in MVI patients was higher than that in non-MVI patients, but there was no such pattern for hepatitis B surface antigen (HBsAg). In terms of liver function, there was no significant difference between the two groups in the comparison of total bilirubin (T-BIL) and alanine transaminase (ALT) (*p* = 0.1011 and 0.1241) and there were significant differences in the comparison of aspartate transaminase (AST) levels (*p* = 0.0362). The MVI-positive patients were generally higher than the MVI-negative patients.

### 3.3. Segmentation Results and Model Comparisons

All CT slices of 71 patients (1861 samples in total) were manually annotated for training and testing, and the trained model automatically segmented the slices of all patients to obtain the ROIs. During this process, the lightweight transformers and other comparison models were executed for 100,000 iterations, and finally, they all converged. The loss value of the validation set remained constant, but there was no overfitting phenomenon. The key hyperparameters were optimized during training. For convenience of the performance comparisons, we set some parameters of each model to be consistent, as shown in Table 2. The optimizer adopted momentum and the momentum factor was set to 0.9.

**Figure 5:**
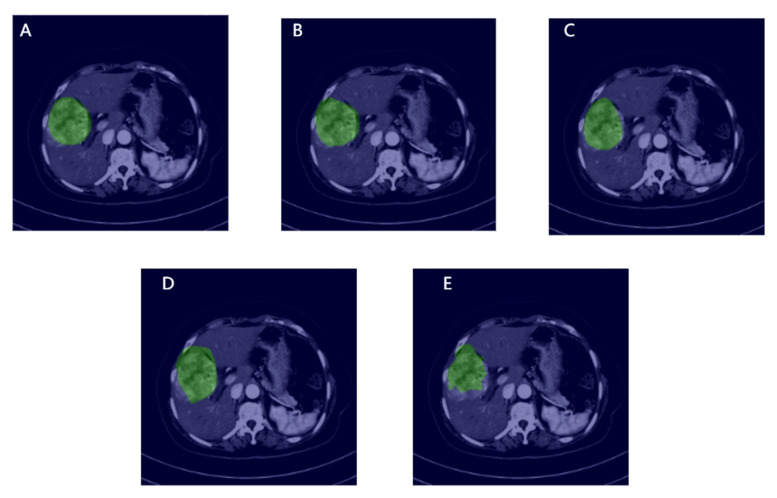
Visualization of manual annotation and segmentation of each model, in which the green area is the ROI, and the rest are the original slices: (**A**) Represents manual annotation; (**B**–**E**) represent MVI-Mind, Swin Transformers, DeepLab V3+, U-Net segmentation, respectively.

The trained models were selected for testing. Figure 5 shows the segmentation criteria and visualization results for each model. The green area represents the ROI that was either manually annotated or considered by the model. From the visualization results, the segmentation effect of the proposed transformer is the closest to that of the standard, followed by the Swin transformer, which highlights the superiority of the transformer architecture in the field of HCC segmentation. The effects of DeepLab V3+ and U-Net are not particularly satisfactory. Table 3 shows the evaluation of the performance of each model. It can be found that MVI-Mind performs the best, where mIoU is 0.9006, Acc is 0.9947, Kappa is 0.8903, and Dice is 0.9451. The Swin transformer performance is similar, with an mIoU of 0.8971. The performances of the baseline models were significantly different from that of the former. DeepLab V3+ and U-Net only had mIoU values of 0.7778 and 0.7521, respectively.

Table 4 compares the number of parameters, total training time, and convergence time of all models. Although the performance of the Swin transformer is not significantly different from that of MVI-Mind, the number of parameters, training time, and convergence time are all larger than those of MVI-Mind. In particular, the convergence time is nearly three times that of the proposed method, which indicates that the training of the Swin transformer is difficult under the same conditions. The baseline models have lower convergence times than the transformer architecture owing to their architectures.

### 3.4. MVI Prediction Results and Models Comparison

The CNN was selected as the MVI prediction model, which can automatically extract the texture features of the ROI in depth and can predict the MVI. This study adopted a 5-fold cross-validation method, and each fold was iterated until the model converged. This was repeated five times, and the mean and 95% CI were calculated. During training, we selected the Adam optimizer, and the key hyperparameters of each model were optimized (for convenience of comparison, some parameter settings of the models were set to be consistent, as shown in Table 5). The verification results of the patients are presented in Table 6. It can be seen that MVI-Mind has the best performance, and the evaluation indicators of the three scanning periods are better than those of other deep learning models with the highest Prec of 0.8750 and F_1_ of 0.8488. ResNet-34 outperformed Inception V3, which could be related to its model architecture. Interestingly, the results of MVI-Mind and ResNet-34 showed that AP predicted the best.

**Figure 6:**
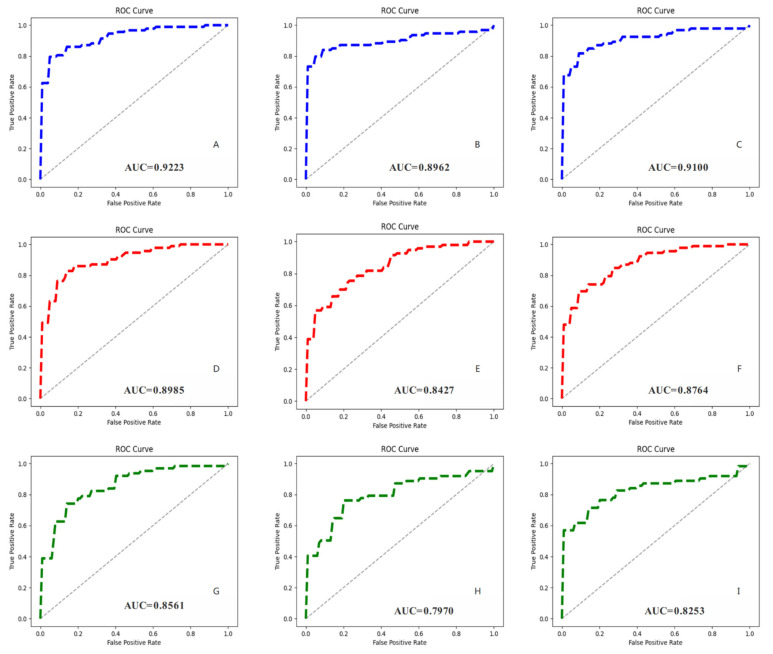
Receiver operating characteristic curves (ROCs) of all CNN models and their corresponding AUC values. (**A**–**C**) Represent the prediction results of MVI-Mind during AP, PVP, DP, respectively; (**D**–**F**) represent the prediction results of ResNet-34 during AP, PVP, DP, respectively; (**G**–**I**) represent the prediction results of Inception V3 during AP, PVP, and DP, respectively.

To reflect the performance of each model more intuitively, we plotted the ROC curves for different periods and calculated the corresponding AUC values (Figure 6). MVI-Mind had the highest AUC, which was 0.9223 for AP, 0.8962 for PVP, and 0.9100 for DP, indicating that the method proposed in this paper has the best generalization ability. This was followed by ResNet-34 (with the highest AUC of 0.8985), and Inception V3 (with the highest AUC of 0.8561).

### 3.5. The End-to-End Prediction Pipeline of MVI-Mind

**Figure 7:**
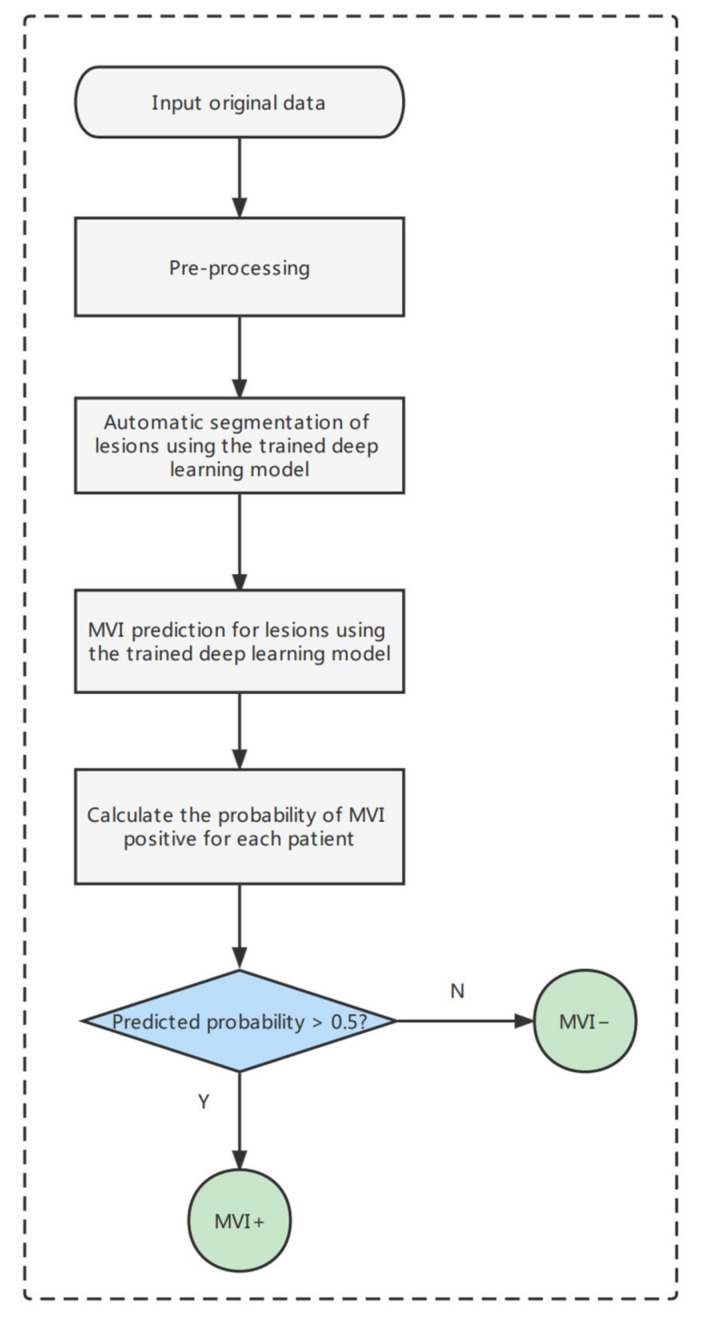
End-to-end prediction pipeline of MVI-Mind.

The MVI-mind decision process based on the trained segmentation and prediction model was designed (Figure 7). After inputting the original CT data of each patient, it is preprocessed to obtain slices first, and then automatically segmented to obtain ROIs. The prediction model is then input to automatically extract features for the probability prediction for each patient according to the clinical decision guideline. If the predicted probability exceeds the threshold, the MVI is considered to be positive; otherwise, it is considered to be negative. The process is fully automated and batched, and it can perform end-to-end MVI prediction using certain scanning period (AP, PVP, or DP) CT images of HCC patients in a short time (the prediction speed reaches 45.8–62.4 s/person in this computing device).

## 4. Discussion

In this paper, for the first time, we proposed MVI-Mind, an end-to-end deep-learning method, integrating image preprocessing, automatic segmentation, automatic feature extraction, and prediction for MVI prediction in HCC patients. Using imaging to efficiently and accurately assess the presence of MVI before surgery would help doctors to make better clinical decisions. A lightweight transformer was adopted to automatically segment liver tumors and their surrounding ROIs, and an mIoU of 0.9006 was achieved. A CNN model was also designed to automatically extract segmented ROI features and accurately predict MVI, with the highest AUC of 0.9223. The results show that the proposed method outperforms current mainstream models.

Among the segmentation modules, owing to the superiority of its architecture, our proposed transformers achieved the best performance. First, the encoder did not use the traditional positional embedding and instead added a 3 × 3 convolution kernel to the feed-forward network, which could better transmit positional information while avoiding performance degradation. Patch embedding added an overlap operation, which was beneficial for enhancing local continuity. Based on traditional self-attention, we added the hyperparameter sr_ratio to control the size of the parameter matrix, thereby, making self-attention more efficient. The decoder designed multiple MLPs to aggregate the information of different layers, thus, combining local and global attention, while greatly reducing the model parameters, thus, further reducing the weight of the model. These improvements further highlight the superiority of transformers in liver ROI segmentation tasks. The model splits the image into patches and maps them into a sequence of linear embeddings encoded by an encoder. This method captures the contextual information of images better than a CNN. Moreover, the number of model parameters is greatly reduced as compared with traditional transformers, and thus, the training difficulty is reduced.

A CNN architecture with four convolutional layers, four pooling layers, and two fully connected layers was designed for prediction, which employed the ReLU activation function and added a dropout to prevent overfitting. The results show that the above CNN performs excellently in predicting the MVI task, even surpassing mainstream models of medical image classification, such as ResNet-34 and Inception V3. Although the proposed CNN architecture is simple, the 4-layer convolution kernel can deeply extract liver ROI features and can achieve an accurate prediction of MVI, and the appropriate number of network layers is not prone to overfitting. In contrast, the network complexity and depth of ResNet-34 and Inception V3 were much higher than those of the aforementioned CNN, but the effect was not as good as that of the latter. A possible reason for this is that the extracted features are too deep owing to the complexity of the model, which leads to overfitting in the classification stage. On the one hand, the texture features of the segmented HCC lesion slices are not complicated as compared with others, such as the mirror image of skin cancer. On the other hand, the small amount of data (138 patients) may also lead to overfitting of the complex models. Therefore, it is very important to design a suitable deep learning model based on the characteristics of the image and amount of data.

During AP, the contrast agent passes through the human arterial blood vessels, and therefore, the arterial blood vessels and the tissues, organs, and lesions rich in arterial blood vessels appear to enhance imaging. In patients with primary HCC, the arterial blood supply of the lesions is rich; therefore, when performing liver-enhanced CT examination for AP, the lesions often show obvious enhancement, and the contrast agent flows out rapidly with the arterial blood. In this study, it was found that the model effect during AP was generally better than that during PVP and DP, and the reasons and application scenarios need to be further discussed in the future.

In recent years, radiomics studies based on deep learning have mostly used 3D methods, that is, 3D segmentation or 3D classification [23,25], but there are also studies that have chosen 2D methods [26]. MVI-Mind designed a 2D input channel, which converted CT images into slices in the preprocessing module, and finally summarized the slice results and predicted the presence of MVI in HCC patients before surgery, according to clinical decision guidelines. There are two reasons for this finding. First, the number of data studied is only 138 cases. If a 3D method was adopted, the amount of data for the segmentation and classification model would be extremely low, which would lead to the failure of the model to effectively learn the data features. In addition, a transformer model was employed for image segmentation. If it was changed to a 3D input channel, the number of parameters would be significantly increased, which would place extremely high demands on the training equipment. Based on the analysis above, the 2D method is more suitable for the actual situation in this study.

Compared with previous similar reports, Jiang et al. [23] included 405 patients for their study. They extracted 7302 radiomics features for lesions and developed machine learning models and 3D-CNN based on radiomics features and clinical baseline data to predict the presence of MVI. The results showed that the AUCs of the machine learning and 3D-CNN models on the validation set were 0.887 and 0.906, respectively, which were both lower than those in our study. Zhang et al. [25] also developed a 3D-CNN to predict MVI in 237 patients with HCC, achieving an AUC of 0.81, a recall of 0.69, and a specificity of 0.79. Obviously, this result was inferior to our work. Yang et al. [40] studied 283 HCC patients, extracted lesion features through a CNN, and integrated radiomics and clinical features for preoperative identification of MVI status, with the highest AUC of 0.909. In [41], the radiology images of 160 patients with HCC were manually segmented into ROIs, after which the authors trained a supervised learning model for predicting MVI with the highest AUC of 0.85 and specificity of 0.762. In this study, only 138 patients were selected, but the highest AUC achieved was 0.9223, which indicates the best performance of the MVI-Mind.

This study had certain shortcomings. For example, the lack of multicenter imaging data has led to further validation of the applicability of this method. The 2D approach adopted also has limitations because segmenting and predicting lose spatial information between slices, which may affect the model’s decision-making performance. The study only built models through radiomics without considering the clinical data of patients, which improved the convenience of practical application but might also lose accuracy. Moreover, the small sample size is also a limitation, as it does not reflect the generalization ability of the proposed method on other patients.

## 5. Conclusions

A traditional diagnosis of MVI requires postoperative pathological detection. In this study, we proposed an end-to-end deep learning strategy based on CT radiomics, which could quickly preprocess raw data, automatically segment the ROI of the liver, automatically extract relevant features, and achieve accurate prediction of MVI. In the segmentation module, the proposed lightweight transformers achieved an mIoU of 0.9006, outperforming other deep-learning algorithms. The prediction results show that the AP works best, and the accuracy of the designed CNN is 0.8678, surpassing that of the mainstream model. In the future, the dataset will be expanded further to verify the generality of the method and apply it to clinical practice.

## Figures and Tables

**Table 1:** Statistics of clinical indicators of datasets employed in the study.

Clinical Indicator	Total Dataset (*n* = 138)		
	MVI Positive (*n* = 68)	MVI Negative (*n* = 70)	
Gender			
Male	56 (82.35%)	57 (81.43%)	
Female	12 (17.65%)	13 (18.57%)	
Age	56.70 ± 11.48	56.34 ± 12.05	*p* = 0.4355
MTD(mm)	5.20 ± 3.48	4.30 ± 1.98	*p* = 0.0321
AFP			
Positive	45 (66.18%)	39 (55.71%)	
Negative	23 (33.82%)	31 (44.29%)	
HBsAg			
Positive	60 (88.24%)	63 (90.00%)	
Negative	8 (11.76%)	7 (10.00%)	
ALB(g/L)	40.79 ± 5.04	40.27 ± 4.81	*p* = 0.2718
T-BIL(mmol/L)	21.43 ± 9.55	17.85 ± 7.60	*p* = 0.1011
ALT(u/L)	63.99 ± 35.78	47.39 ± 25.92	*p* = 0.1241
AST(u/L)	60.70 ± 39.10	34.74 ± 18.35	*p* = 0.0362

Note: MTD, AFP, HBsAg, ALB, T-BIL, ALT, and AST represent maximum tumor diameter, alpha-fetoprotein, Hepatitis B surface antigen, albumin, the total bilirubin, alanine aminotransferase and aspartate aminotransferase, respectively. Additionally, some indicators are represented by the mean values of the samples and the corresponding 95% confidence intervals.

**Table 2:** Configuration of key parameters of lesion segmentation module in MVI-Mind framework.

Parameter Name	Parameter Value
num_classes	2
base_learning rate	0.005
momentum	0.9
weight_decay	4.0 × 10^−5^
batch_size	2

**Table 3:** Performance comparisons of various deep automatic segmentation models.

Model	mIoU	Acc	Kappa	Dice
MVI-Mind (our work)	0.9006	0.9947	0.8903	0.9451
Swin transformer	0.8971	0.9943	0.8860	0.9430
DeepLab V3+	0.7778	0.9871	0.7185	0.8592
U-Net	0.7521	0.9863	0.6758	0.8378

**Table 4:** Performance comparisons of various deep automatic segmentation models.

Model	Num_params	Num_iters	Total Training Time/s	Convergence Time/s
MVI-Mind (our work)	84,596,418	100,000	63,075	about 6550
Swin transformer	108,235,650	100,000	78,420	about 15,680
DeepLab V3+	45,871,090	100,000	40,218	about 3890
U-Net	13,404,354	100,000	18,930	about 1520

**Table 5:** Configuration of key parameters of the MVI prediction module in MVI-Mind framework.

Parameter Name	Parameter Value
num_classes	2
learning_rate	1.0 × 10^−6^
optimizer	Adam
weight_decay	3.0 × 10^−3^
batch_size	64
verbose	1

**Table 6:** The performance of each deep learning model in the MVI prediction task.

Model	Scan Time Period	Acc	Rec	Prec	F_1_ Score
MVI-Mind	AP (avg ± 95%CI)	0.8678	0.8269	0.8750	0.8488
		±0.0458	±0.0767	±0.0490	±0.0566
	PP (avg ± 95%CI)	0.8534	0.7760	0.8972	0.8241
		±0.0484	±0.1060	±0.0651	±0.0645
	DP (avg ± 95%CI)	0.8434	0.7637	0.8823	0.8150
		±0.0547	±0.0802	±0.0816	±0.0660
ResNet-34	AP (avg ± 95%CI)	0.8283	0.6988	0.9089	0.7875
		±0.0242	±0.0372	±0.0676	±0.0303
	PP (avg ± 95%CI)	0.7844	0.6684	0.8313	0.7356
		±0.0474	±0.0905	±0.0732	±0.0688
	DP (avg ± 95%CI)	0.7889	0.6848	0.8271	0.7409
		±0.0653	±0.1242	±0.0834	±0.0919
Inception-V3	AP (avg ± 95%CI)	0.7940	0.7256	0.8061	0.7599
		±0.0269	±0.0738	±0.0478	±0.0439
	PP (avg ± 95%CI)	0.7728	0.6949	0.7911	0.7380
		±0.0525	±0.0450	±0.0787	±0.0512
	DP (avg ± 95%CI)	0.7947	0.7423	0.8133	0.7682
		±0.0501	±0.0793	±0.0948	±0.0494

Note: Each model’s results are the mean of 5 predictions and the corresponding 95% confidence interval.

## Data Availability

Not applicable.
